# TIGIT expression in renal cell carcinoma infiltrating T cells is variable and inversely correlated with PD-1 and LAG3

**DOI:** 10.1007/s00262-024-03773-8

**Published:** 2024-08-06

**Authors:** Oscar Perales, Lucia Jilaveanu, Adebowale Adeniran, David G. Su, Michael Hurwitz, David A. Braun, Harriet M. Kluger, David A. Schoenfeld

**Affiliations:** 1 Yale School of Medicine, New Haven, CT USA; 2 Section of Medical Oncology, Yale School of Medicine, 333 Cedar Street, FMP120, New Haven, CT 06520 USA; 3 Department of Pathology, Yale School of Medicine, New Haven, CT USA; 4 Division of General Surgery, Department of Surgery, Yale School of Medicine, New Haven, CT USA

**Keywords:** TIGIT, Renal cell carcinoma, Kidney cancer, Immune checkpoint inhibitors, LAG3, PD-1

## Abstract

**Purpose:**

Immune checkpoint inhibitors have revolutionized the treatment of renal cell carcinoma (RCC), but many patients do not respond to therapy and the majority develop resistant disease over time. Thus, there is increasing need for alternative immunomodulating agents. The co-inhibitory molecule T-cell immunoglobulin and ITIM domain (TIGIT) may play a role in resistance to approved immune checkpoint inhibitors and is being investigated as a potential therapeutic target. The purpose of this study was to quantify TIGIT positivity in tumor-infiltrating T cells in RCC.

**Methods:**

We employed tissue microarrays containing specimens from primary RCC tumors, adjacent normal renal tissue, and RCC metastases to quantify TIGIT within tumor-infiltrating CD3^+^ T cells using quantitative immunofluorescent analysis. We also compared these results to TIGIT^+^ CD3^+^ levels in four other tumor types (melanoma, non-small cell lung, cervical, and head and neck cancers).

**Results:**

We did not observe significant differences in TIGIT positivity between primary RCC tumors and patient-matched metastatic samples. We found that the degree of TIGIT positivity in RCC is comparable to that in lung cancer but lower than that in melanoma, cervical, and head and neck cancers. Correlation analysis comparing TIGIT positivity to previously published, patient-matched spatial proteomic data by our group revealed a negative association between TIGIT and the checkpoint proteins PD-1 and LAG3.

**Conclusion:**

Our findings support careful evaluation of TIGIT expression on T cells in primary or metastatic RCC specimens for patients who may be treated with TIGIT-targeting antibodies, as increased TIGIT positivity might be associated with a greater likelihood of response to therapy.

**Supplementary Information:**

The online version contains supplementary material available at 10.1007/s00262-024-03773-8.

## Introduction

In renal cell carcinoma (RCC), immune checkpoint inhibitor (ICI)-based regimens have significantly improved overall survival compared to previous standard-of-care tyrosine kinase inhibitors (TKIs). For example, a small minority of patients treated with the combination of ipilimumab (anti-CTLA-4) and nivolumab (anti-PD-1) remain in complete response and off therapy after more than 6 years [1, 2]. However, resistance to ICI-based regimens remains a challenge, highlighting a need for alternative immunotherapeutic strategies.

Other co-inhibitory receptors expressed on immune cells, such as LAG3, TIM-3 and TIGIT, are promising targets [3]. Expression of these co-inhibitory receptors results in increased tumor tolerance and T cell anergy through pathways parallel to the canonical PD-1/PD-L1 and CTLA-4/CD80/86 axes. Engagement of TIGIT on T cells downregulates T cell receptor complex components, impairing recognition of tumor antigens [4]. TIGIT is highly expressed on immunosuppressive regulatory T cells (Tregs) and natural killer (NK) cells, stimulating production of the anti-inflammatory cytokine IL-10 and downregulating tumor surveillance [3, 5]. Previous work has suggested a TIGIT receptor-positive cell density of 0.4% in RCC primary tumors and 2.1% in metastases, notably lower than receptor-positive densities for LAG3 and TIM-3; expression of these co-inhibitory receptors was also largely mutually exclusive and could define distinct tumor subtypes [6]. Single-cell RNA-sequencing studies in RCC have further defined *TIGIT* expression patterns in the tumor microenvironment (TME). One such study demonstrated enrichment of *TIGIT*, as well as other immune checkpoints, on terminally exhausted CD8^+^ T cells in the primary tumors of patients that would go on to develop more advanced disease [7]. TIGIT’s role as part of a posited dysfunctional immune circuit involving T cells and macrophages leading to more advanced disease in RCC was further confirmed in this study through flow cytometry analysis of TIGIT expression on terminally exhausted CD8^+^ T cells paired with expression of CD155, a TIGIT ligand, on M2-like, immunosuppressive macrophages. TIGIT expression thus may serve as a biomarker to predict resistance to immunotherapy or as a therapeutic target itself.

TIGIT blockade is being evaluated in multiple clinical trials, including phase III studies for advanced non-small cell lung cancer (NSCLC) and melanoma, and phase II basket trials for solid tumors, although none treating RCC specifically (NCT04294810, NCT05665595, NCT04693234, NCT03708224). Of note, only patients with elevated PD-L1 expression were found to benefit from TIGIT blockade in one phase II trial [8]. The role of TIGIT expression on CD3^+^ T cells as a predictive biomarker is unknown.

Given the interest in anti-TIGIT therapies in solid tumors, our purpose was to evaluate the degree of TIGIT expression on tumor-infiltrating T cells in RCC at different anatomic sites and relative to other cancer types. We performed immunofluorescent analysis of TIGIT positivity on CD3^+^ T cells on cohorts of primary RCC tumors and matched adjacent normal kidney samples, matched RCC primary and metastatic tumors, and cohorts from four other tumor types (melanoma, NSCLC, cervical, and head and neck cancer). Overall, we found no differences between RCC primary tumors and metastases and TIGIT positivity in RCC was comparable to NSCLC but lower than in the other cancer types.

## Materials and methods

### Tissue microarrays

We performed these studies using two previously reported RCC tissue microarrays (TMAs) (Table 1) [9–13]. YTMA84-A1 and YTMA84-B1 contained primary tumor tissue and adjacent normal renal parenchyma, and YTMA166-1 contained paired primary tumors and metastases. The TMAs consisted of 0.6 mm cores spaced 0.8 mm apart. Our positive control YTMA462 contained lymphoid tissue from tonsil and spleen (*n* = 11). YTMA226-5 was used for lung cancer (*n* = 22), YTMA383-1 for cervical cancer (*n* = 23), YTMA335-8 for melanoma (*n* = 13), and YTMA275 for head and neck cancer (*n* = 27).

**Table 1:** Tissue microarray clinical characteristics

	YTMA84	YTMA166
Patients	205	57
Matched pairs	20	15
Total samples*	284	168
Sample type		
Primary tumor	230	87
Metastasis	–	81
Normal kidney	54	–
Sex		
Male	137	33
Female	68	24
Age in years (SD)	62.5 (13.2)	54.3 (13.3)
Tumor size (SD)**	6.0 cm (3.5)	7.2 cm (3.4)
Histology		
ccRCC	143	46
nccRCC	46	3
Stage		
1	112	–
2	17	–
3	55	–
4	20	57
Grade**		
1	22	1
2	108	23
3	56	23
4	18	1

Clinical and pathologic characteristics of patient samples from YTMA84 and 166 used in this analysis. Due to missing or incomplete information for some patients, all values may not sum to total

*SD* standard deviation, *ccRCC* clear cell RCC, *nccRCC* non-clear cell RCC

^*^Includes all biologic replicates

^**^Based on primary tumors only

### Immunostaining, multispectral image acquisition and analysis

Briefly [14], mouse anti-CD3 antibody (Invitrogen MA5-12577) was diluted 1:100 and incubated overnight at 4 °C. Signal amplification was performed using anti-mouse EnVision antibody-HRP (Dako K4001) and HRP-activated-Cy5-tyramide (1:50; Akoya Biosciences) following the manufacturer’s protocol. HRP-quenching was done with 100 mM benzoic hydrazide + 50 mM hydrogen peroxide. Slides were incubated for 2 h at room temperature with anti-TIGIT antibody (Abcam 243903) diluted 1:400. Anti-TIGIT signal was amplified using anti-rabbit EnVision system-HRP (Dako K4003) and Cy3-Tyramide (1:50; Akoya Biosciences). Slides were incubated with 4,6-diamidino-2-phenylindole (DAPI) and mounted with ProLong mounting medium (ProLong Gold; Invitrogen, Carlsbad, CA, USA).

Monochromatic image acquisition was performed using Aquasition (HistoRx) software on images captured through a microscopy-based multiplex imaging device and microarray reader (PM-3000; HistoRx). Due to generally low levels of TIGIT positivity on CD3^+^ cells on the RCC TMAs, cell counts were manually quantified. On slides with high density of CD3 staining, cell counts were extrapolated from representative quadrants. For this study, Cy3^+^, Cy5^−^ cells were excluded from analysis.

### Statistics

Statistical analysis and graphing were performed using GraphPad Prism 10 (GraphPad Software, Inc.). TCGA data were taken from cBioPortal [15–17]. Some tumor samples on YTMA166 were represented by multiple biologic replicates; when present, replicate CD3 counts and levels of TIGIT positivity were averaged. Kruskal–Wallis test with Dunn’s test for multiple comparisons was used to compare TIGIT positivity levels between tumor types. Mann–Whitney tests were used for testing mean differences between samples. For matched-pair analysis, Wilcoxon matched-pair test was used. ANOVA was used to compare TIGIT^+^ T cells across metastatic sites. Chi-squared test was used to test differences in TIGIT positivity across tumor types. Correlation analyses were performed using Spearman’s rank test; outliers were detected and removed with formal outlier testing using Grubb’s test with a *P* < 0.01 considered significant. All other statistical testing was performed with a two-sided *P* < 0.05 considered significant.

## Results

### High *TIGIT* expression in RCC is associated with increased tumor grade, stage, and decreased survival

**Fig. 1:**
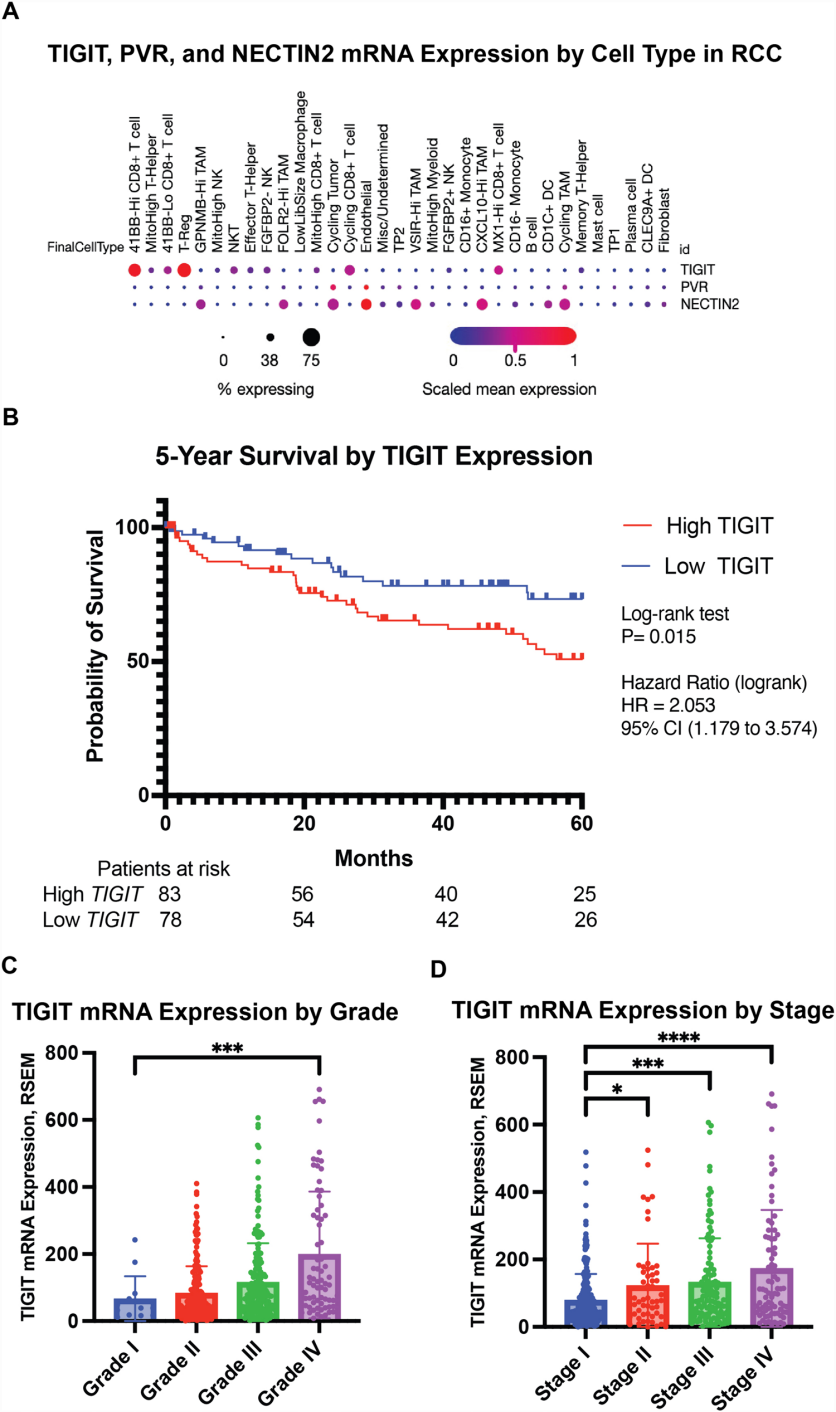
**A** Dot plot of single-cell mRNA expression for *TIGIT*, *PVR*, and *NECTIN2* in the RCC TME*,* by cell type, from the Broad Institute’s Single Cell portal and [18]. **B** Kaplan–Meier curves of overall survival for ccRCC patients based on level of *TIGIT* mRNA expression, with high *TIGIT* expressors having a z-score > 1, and low *TIGIT* expressors having a z-score <  − 1. Expression and survival data from the TCGA PanCancer Atlas on cBioPortal. *TIGIT* mRNA expression in ccRCC by** C** histologic grade and **D** stage. Statistical testing was performed for **C, D** using ANOVA Dunnett test for multiple comparisons, with Grade or Stage I as the control. *p < 0.05; **p < 0.01;***p < 0.001; ****p < 0.0001

We used a single-cell transcriptomic dataset to confirm the expression of *TIGIT* and its ligands, *NECTIN2* and *PVR*, in the RCC TME [18]. We found that *TIGIT* is mainly expressed on T cells, while the *TIGIT* ligands, primarily *NECTIN2*, are expressed on tumor-associated macrophages (TAMs), tumor cells, and endothelial cells, in line with findings from other RCC single-cell RNA studies (Fig. 1A) [7]. We then queried publicly available TCGA PanCancer genomic datasets on cBioPortal to compare *TIGIT* mRNA expression in RCC with other solid malignancies [15–17]. We found that expression of *TIGIT* was relatively high in clear cell RCC (ccRCC), but lower than in other malignancies, including melanoma, NSCLC, head and neck, and cervical cancer (Supplementary (Supp.) Fig. S1A). With prior studies demonstrating a relationship between TIGIT expression and the development of more advanced disease at the single-cell level but with a relatively limited number of patient samples [7], we next sought to determine the relationship between *TIGIT* expression and overall survival (OS) in ccRCC using the larger TCGA PanCancer dataset. We categorized patients as having either high, intermediate, or low *TIGIT* expression using z-scores [15, 19]. Z-scores > 1 were defined as “high” and < − 1 as “low.” Comparing high to low *TIGIT* expressors, high *TIGIT* expression was associated with worse 5-year OS (HR = 2.05, *p* = 0.015) (Fig. 1B). Advanced histologic grade and stage were also associated with higher *TIGIT* levels, in accord with the single-cell transcriptomic and flow cytometry findings from the prior study (Fig. 1C, D) [7]. However, a Cox proportional hazards model incorporating *TIGIT* levels, grade, and stage showed that *TIGIT* expression may not be an independent prognostic factor (Supp. Table S1).

### TIGIT positivity is similar between RCC primary tumors and metastases

**Fig. 2:**
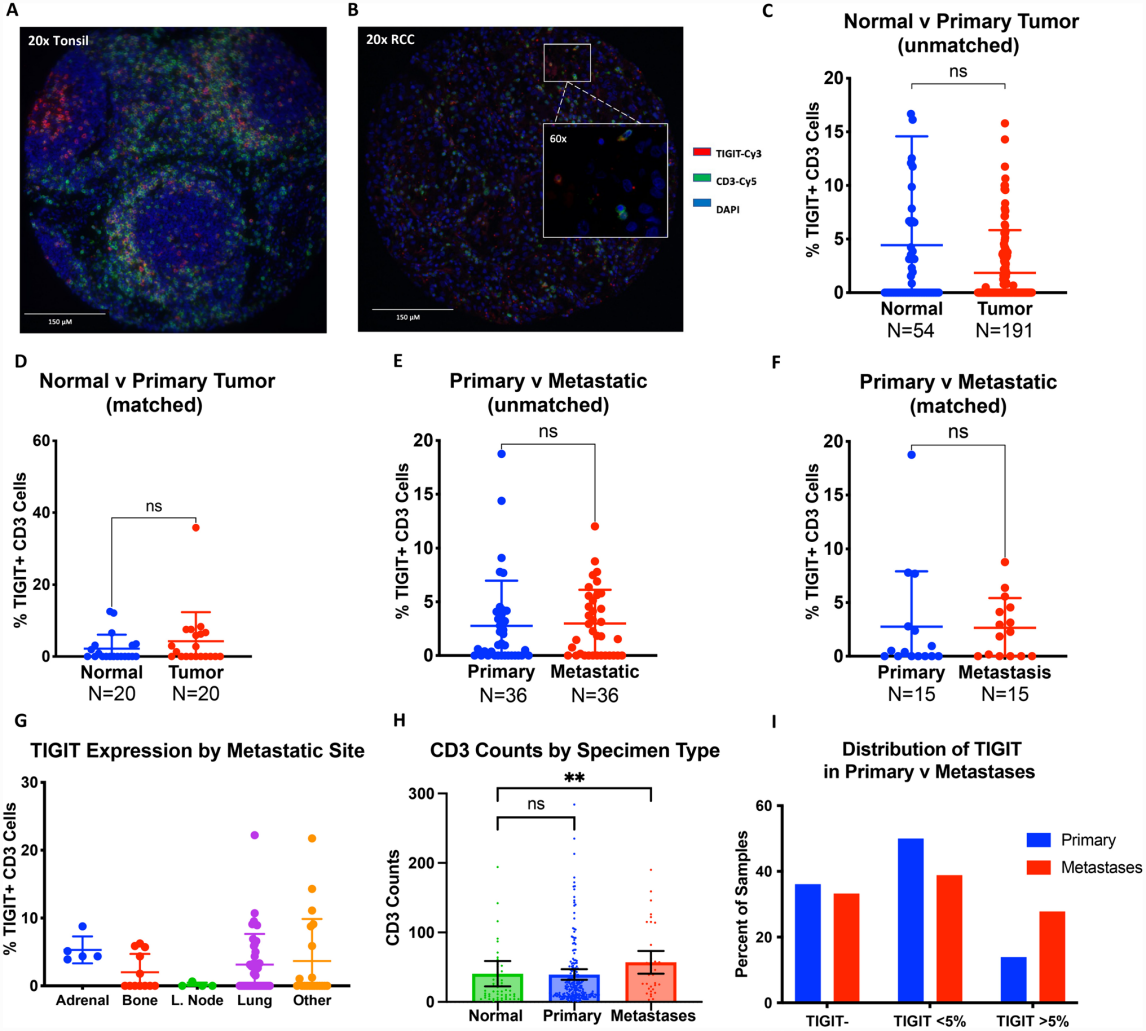
**A** Exemplary immunofluorescent staining of TIGIT and CD3 in a tonsillar tissue specimen, which served as a positive control. **B** Representative stain of TIGIT and CD3 in RCC primary tumor tissue. Percentage of CD3 + cells expressing TIGIT in: **C** unmatched and **D** matched adjacent normal renal tissue and primary RCC tumors from YTMA84; and **E** unmatched and **F** matched RCC primary tumors and metastases from YTMA166; and** G** multiple anatomic sites of RCC metastases from YTMA166. **H)** Absolute CD3 + cell counts in adjacent normal renal tissue, RCC primary tumors, and metastases, with mean and 95% CI graphed. **I** Distribution of high (≥ 5%), low (< 5%), and absent TIGIT positivity levels comparing unmatched primary RCC tumors versus metastases from YTMA166. Statistical tests performed with T test or ANOVA. *p < 0.05; **p < 0.01; ***p < 0.001

We quantified the percentage of T cells (CD3^+^) that were TIGIT-positive in RCC through immunofluorescence. As TIGIT is highly expressed in tonsillar tissue, we employed a TMA of normal tonsil as a positive staining control and to establish the staining conditions (Fig. 2A) [20]. An example of TIGIT-positive staining on T cells in an RCC TMA is shown in Fig. 2B.

We compared the percentage of TIGIT-expressing T cells between primary RCC tumors, adjacent normal tissue, and metastatic sites. There were no differences between normal adjacent renal tissue (*n* = 54) and RCC primary tumors (*n* = 191) in unmatched and matched-pair (*n* = 20) patient samples (Fig. 2C, D). Likewise, the density of TIGIT^+^ T cells was similar between primary (*n* = 36) and metastatic (*n* = 36) tumor tissue in unmatched as well as matched-pair (*n* = 15) analysis (Fig. 2E, F). TIGIT positivity was also not significantly different at various RCC anatomic sites of metastasis (Fig. 2G). Among clinical factors, we found a significant but weak correlation with tumor size (*R*^2^ = 0.062, *p* = 0.006) (Supp. Fig. S2). Additionally, RCC metastases had significantly higher levels of T cell infiltration compared to normal renal parenchyma, but not relative to primary tumor specimens (Fig. 2H). RCC primary tumors and metastases had similar percentages of TIGIT-negative samples (*p* = 0.805) (Fig. 2I). Among the TIGIT^+^ samples (TIGIT^+^ T cells > 0%), we further dichotomized based on degree of TIGIT T cell positivity, defining high expressors as having ≥ 5% positive T cells and low expressors as having < 5%. Still, the proportion of high expressors was comparable in primary tumors and metastases (*p* = 0.332).

### TIGIT positivity on tumor-infiltrating T cells is comparable between RCC and NSCLC, but higher in other tumor types

**Fig. 3:**
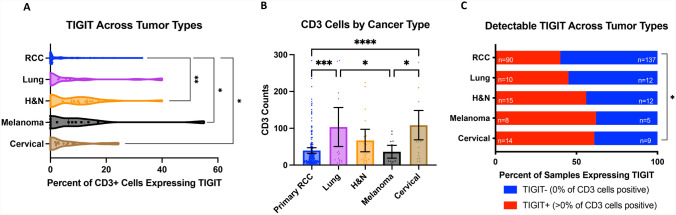
**A** Violin plots showing percentage of TIGIT + CD3 cells across multiple tumor types. **B** Absolute CD3 + cell counts across RCC primary tumor samples and the other indicated cancer types. **C** Percentage of TIGIT + (> 0%) samples in each of the indicated cancer types. RCC bar includes primary tumors from YTMA84 and YTMA166. Significance tested by Chi-squared test. *p < 0.05; **p < 0.01; ***p < 0.001, ****p < 0.0001

Given the high levels of *TIGIT* mRNA expression in melanoma, NSCLC, head and neck, and cervical cancer, we also stained TMAs containing these tumor types for CD3 and TIGIT. The percentage of T cells expressing TIGIT was significantly higher in melanoma, cervical, and head and neck cancer relative to RCC primary tumors (Fig. 3A). While the measurement of TIGIT positivity is intrinsically normalized to T cell density, we additionally quantified absolute T cell counts across tumor types. We observed lower degrees of T cell infiltration in RCC compared to NSCLC and cervical cancer, which had the highest T cell counts among the tumor types evaluated (Fig. 3B). Across RCC primary tumors, 39.6% of samples had any level of TIGIT^+^ T cells (i.e., TIGIT^+^ T cells > 0%) (Fig. 3C). Melanoma and cervical cancer had the highest proportion of TIGIT^+^ tumors at > 60%, although only cervical cancer had a significantly higher degree of positivity compared to RCC (*p* = 0.0491).

### TIGIT positivity is negatively correlated with PD-1 and LAG3 protein expression in RCC

**Fig. 4:**
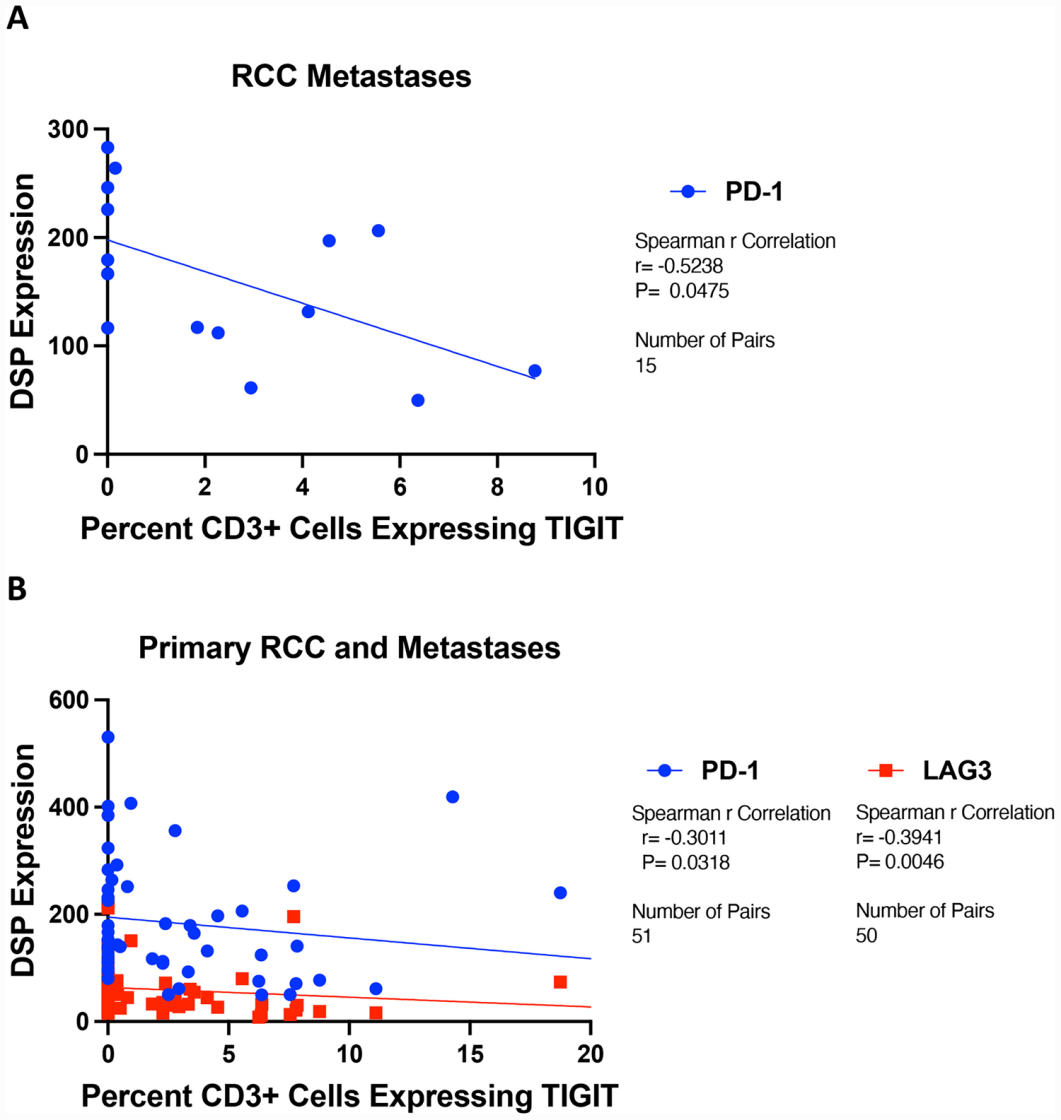
Percentage of TIGIT + CD3 cells graphed against patient-matched spatial proteomic profiling of the indicated immune cell (CD45 +) markers in **A** RCC metastases, and **B** all RCC tumor samples. Spearman rank test was used to assess the correlation between percentage of TIGIT + CD3 cells and each immune marker. Best fit linear regression lines are shown

We previously performed spatial proteomic profiling on overlapping samples from the RCC TMAs to characterize expression patterns of multiple tumor immunomarkers, including CD3, PD-1, PD-L1, LAG3, TIM-3, and CTLA-4 in immune cells (CD45^+^), and PD-L1 in tumor cells (CK^+^) [9, 10]. To better understand the relationship between TIGIT and these other immune proteins, we performed Spearman tests for correlation between the degree of TIGIT positivity and these markers, looking separately at primary tumors, normal adjacent renal tissue, and metastases. Our manual CD3 counts correlated with the degree of CD3 expression as determined by the digital spatial profiling method (*p* = 0.019, Supp. Fig S3). There were no significant correlations between TIGIT^+^ T cell counts and these markers in primary tumors and normal renal tissue (Supp. Fig. S4A, B). Among metastases only, there was a negative correlation with PD-1 expression (*r* = −0.524, *p* = 0.048), and among all tumor samples (primary tumors and metastases), there were negative correlations between TIGIT and PD-1 (*r* = −0.301, *p* = 0.032), and TIGIT and LAG3 (*r* = −0.394, *p* = 0.005) (Fig. 4A, B and Supp. Fig S4C, D).

## Discussion

Our analysis aimed to better characterize expression patterns of the immune checkpoint protein TIGIT on tumor-infiltrating T cells in RCC. Using TCGA PanCancer transcriptomic data, we found that *TIGIT* mRNA levels are relatively high in ccRCC compared to most other tumor types, and that *TIGIT* expression was positively correlated with higher histologic grade and stage, in accord with prior single-cell transcriptomic findings. Immunofluorescent studies showed that TIGIT was detectable on tumor-infiltrating T cells in ~ 40% of RCC patient samples, although only a small subset had ≥ 5% TIGIT-positive T cells. While the degree of TIGIT positivity was significantly higher in melanoma, head and neck, and cervical cancer compared to RCC, there was no significant difference in TIGIT^+^ T cell infiltration between RCC and NSCLC, a tumor type where anti-TIGIT therapies are being extensively studied with numerous late-stage clinical trials underway [21].

Additionally, we found no significant differences between primary RCC tumors and metastases, and between different metastatic sites. We note that many patients with metastatic RCC might only have previously resected large primary tumor samples available for tissue analysis. Our data suggest that sampling from either primary or metastatic sites may be sufficient to determine TIGIT T cell positivity.

Ongoing clinical trials are investigating anti-TIGIT agents in combination with other ICIs based partially on the hypothesis that upregulation of TIGIT may serve as a mechanism of resistance to first-line ICIs. We found a negative correlation between TIGIT positivity on T cells and PD-1 and LAG3 protein levels on tumor-infiltrating immune cells, supporting the hypothesis that TIGIT upregulation may compensate for low expression levels of other immune checkpoint proteins in RCC. These findings are also in agreement with previous research noting the presence of phenotypically distinct tumor clusters, where TIM-3, LAG3 and TIGIT expression in the RCC TME appear to be mutually exclusive [6]. This study further showed that each cluster may be associated with distinct genomic alterations, notably p53/cell cycle alterations in LAG3-positive tumors. Further studies, such as single-cell spatial multi-omic investigation of patient samples pre- and post-ICIs, are needed to help elucidate the molecular drivers regulating the expression of specific co-inhibitory receptors in the RCC TME.

One limitation of our study is that we focused on TIGIT expression on CD3^+^ T cells, while there are other immune populations that express this protein, such as NK cells. Future studies should focus on determining the expression of TIGIT in other immune cell types in RCC, including NK cells, and aim to further characterize expression in T cell subsets, including cytotoxic CD8^+^, CD4^+^, and Treg cells. We also note that the percentage of PD-L1 expressing cells in melanoma and RCC is low compared to lung cancer, yet response rates to PD-1/L1 inhibitors are higher in melanoma and RCC [22]. Similar to PD-L1 positivity as a predictive biomarker for PD-1/L1 inhibitors, TIGIT positivity might not correlate with response to anti-TIGIT antibodies.

In summary, we quantified the percentage of TIGIT-positive T cells in RCC tumors across different anatomic sites of disease and compared to other tumor types. We detected some level of TIGIT positivity in ~ 40% of RCC specimens, comparable to NSCLC, but lower than melanoma, head and neck, and cervical cancer cohorts. Additionally, the percentage of T cells expressing TIGIT in RCC metastatic samples was similar to primary tumors, indicating that sampling from either site is sufficient to determine TIGIT T cell positivity. As ongoing clinical trials investigating anti-TIGIT agents in other malignancies proceed, the results of this study suggest that TIGIT blockade may need to be selectively employed in patients with RCC.

## Supplementary Information

Below is the link to the electronic supplementary material.

Supplementary file1 (PDF 893 KB)

Supplementary file2 (DOCX 97 KB)

## Data Availability

The dataset upon which this study is based is available upon reasonable request.
