# Supplementary material for: TIGIT expression in renal cell carcinoma infiltrating T cells is variable and inversely correlated with PD-1 and LAG3
Source: Cancer Immunol Immunother. 2024 Aug 6;73(10):192. doi: 10.1007/s00262-024-03773-8 (PMC11303630; doi:10.1007/s00262-024-03773-8)
Supplement: Supplementary file 1 — Supplementary file1 (PDF 893 KB) [file 262_2024_3773_MOESM1_ESM.pdf]

Supplementary Figure 1

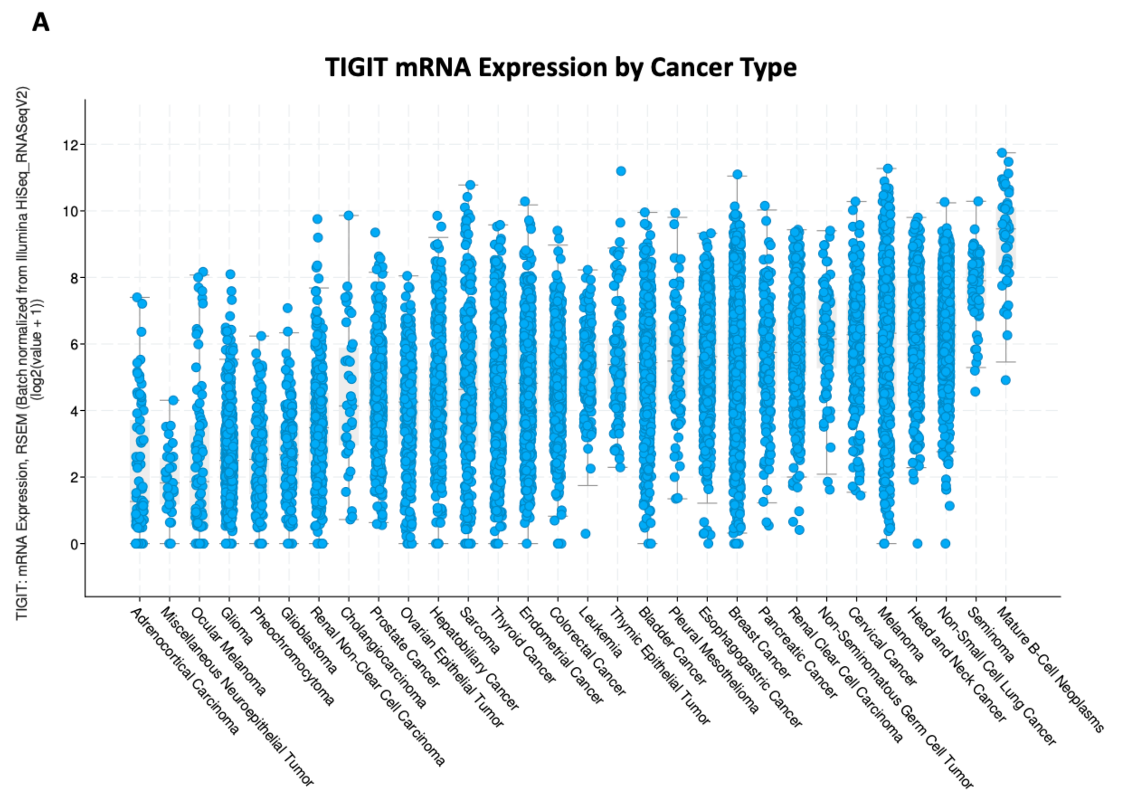

Supp. Figure S1: A) *TIGIT* mRNA expression data from TCGA PanCancer Atlas study, available on cBioPortal. Expression is listed in ascending order based on median, for multiple cancer types.

Supplementary Figure 2

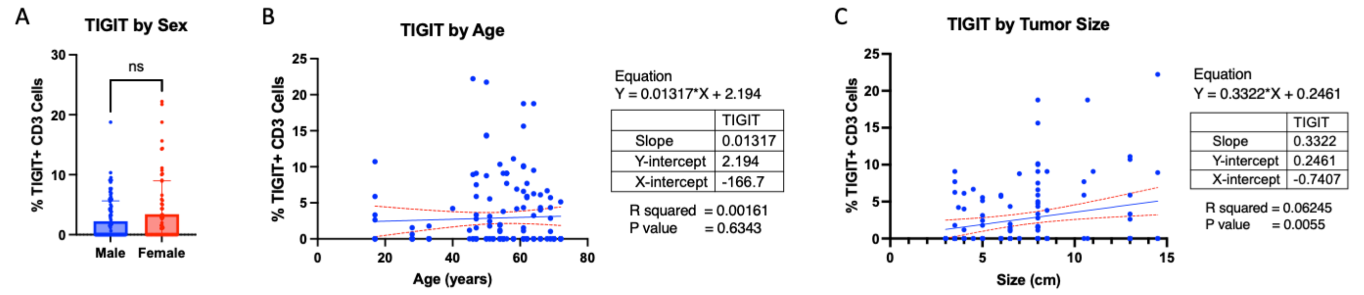

Supp. Figure S2: A-C) Percentage of CD3+ cells expressing TIGIT in RCC plotted against multiple clinical parameters, including patient sex, age, and tumor size. Linear regression models included for the relationship between T cell TIGIT positivity and age and tumor size.

Supplementary Figure 3

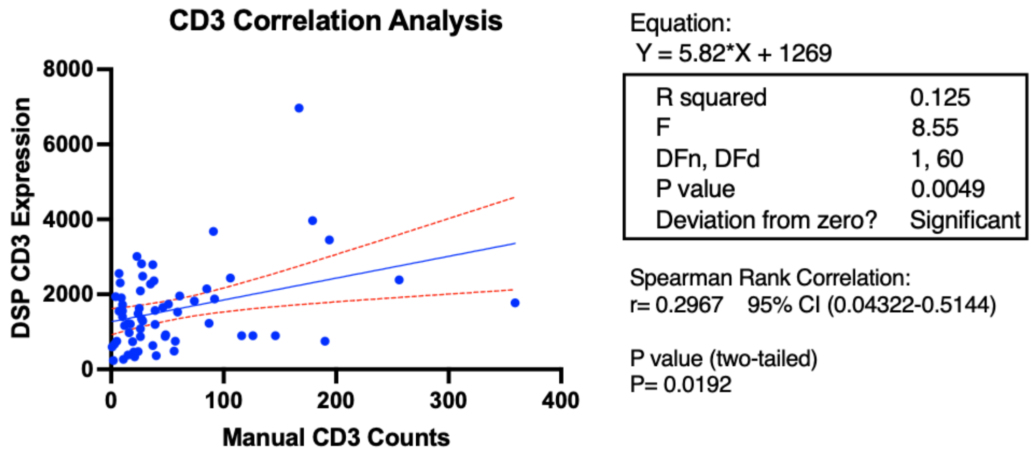

Supp. Figure S3: Linear regression model with 95% CI and Spearman rank correlation test of manual CD3 counts from RCC TMA using IF staining and CD3 expression in CD45+ compartment of digital spatial profiling (DSP) analysis. Samples analyzed included RCC primary and metastatic tumor specimens, and normal kidney parenchyma.

Supplementary Figure 4

A

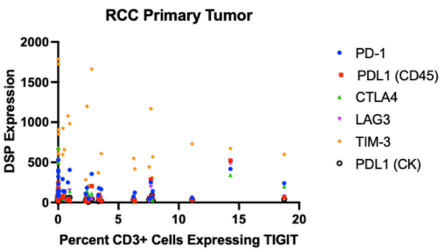

	TIGIT vs. PD-1	TIGIT vs. PDL1 (CD45)	TIGIT vs. CTLA4	TIGIT vs. LAG3	TIGIT vs. TIM-3	TIGIT vs. PDL1 (CK)
Spearman r						
r	-0.2118	-0.02600	-0.1311	-0.3094	-0.1333	0.1275
95% confidence interval	-0.5127 to 0.1354	-0.3603 to 0.3143	-0.4487 to 0.2160	-0.5857 to 0.03137	-0.4505 to 0.2138	-0.2417 to 0.4644
P value						
P (two-tailed)	0.2149	0.8804	0.4462	0.0663	0.4383	0.4869
P value summary	ns	ns	ns	ns	ns	ns
Exact or approximate P value?	Approximate	Approximate	Approximate	Approximate	Approximate	Approximate
Significant? (alpha = 0.05)	No	No	No	No	No	No
Number of XY Pairs	36	36	36	36	36	32

B

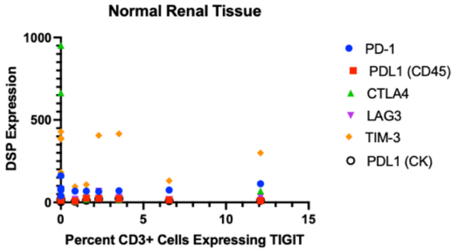

	TIGIT vs. PD-1	TIGIT vs. PDL1 (CD45)	TIGIT vs. CTLA4	TIGIT vs. LAG3	TIGIT vs. TIM-3	TIGIT vs. PDL1 (CK)
Spearman r						
r	0.3024	-0.03257	-0.3862	0.07444	0.1349	-0.03722
95% confidence interval	-0.3810 to 0.7721	-0.6328 to 0.5921	-0.8078 to 0.2969	-0.5641 to 0.6573	-0.5210 to 0.6906	-0.6356 to 0.5890
P value						
P (two-tailed)	0.3621	0.9277	0.2387	0.8290	0.6903	0.9168
P value summary	ns	ns	ns	ns	ns	ns
Exact or approximate P value?	Exact	Exact	Exact	Exact	Exact	Exact
Significant? (alpha = 0.05)	No	No	No	No	No	No
Number of XY Pairs	11	11	11	11	11	11

C

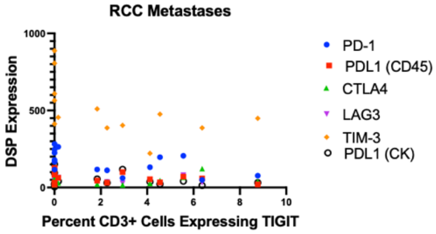

	TIGIT vs. PD-1	TIGIT vs. PDL1 (CD45)	TIGIT vs. CTLA4	TIGIT vs. LAG3	TIGIT vs. TIM-3	TIGIT vs. PDL1 (CK)
Spearman r						
r	-0.5238	-0.1014	0.1807	-0.3209	-0.2268	0.02213
95% confidence interval	-0.8224 to 0.0009917	-0.5943 to 0.4468	-0.3798 to 0.6442	-0.7236 to 0.2448	-0.6715 to 0.3378	-0.5083 to 0.5404
P value						
P (two-tailed)	0.0475	0.7170	0.5152	0.2411	0.4121	0.9391
P value summary	*	ns	ns	ns	ns	ns
Exact or approximate P value?	Exact	Exact	Exact	Exact	Exact	Exact
Significant? (alpha = 0.05)	Yes	No	No	No	No	No
Number of XY Pairs	15	15	15	15	15	15

D

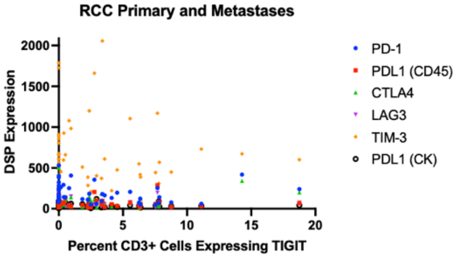

	TIGIT vs. PD-1	TIGIT vs. PDL1 (CD45)	TIGIT vs. CTLA4	TIGIT vs. LAG3	TIGIT vs. TIM-3	TIGIT vs. PDL1 (CK)
Spearman r						
r	-0.3011	-0.1128	-0.02060	-0.3941	-0.1068	0.1174
95% confidence interval	-0.5385 to -0.01952	-0.3864 to 0.1791	-0.3049 to 0.2671	-0.6113 to -0.1217	-0.3813 to 0.1849	-0.1875 to 0.4017
P value						
P (two-tailed)	0.0318	0.4356	0.8871	0.0046	0.4603	0.4372
P value summary	*	ns	ns	**	ns	ns
Exact or approximate P value?	Approximate	Approximate	Approximate	Approximate	Approximate	Approximate
Significant? (alpha = 0.05)	Yes	No	No	Yes	No	No
Number of XY Pairs	51	50	50	50	50	46

Supp. Figure S4: A-D) Percentage of TIGIT+ CD3 cells graphed against patient-matched spatial proteomic profiling of multiple immune marker proteins, by tissue type: **A)** RCC primary tumors; **B)** adjacent normal renal tissue; **C)** RCC metastases; and **D)** all RCC tumor samples. Spearman test was used to assess correlation between percentage of TIGIT+ CD3 cells and each corresponding immune marker, with analysis table displayed underneath the graph. Spatial proteomic analysis included PD-L1 expression in both immune cells (CD45+) and tumor cells (CK+), both of which are shown separately. All other markers represent expression in immune cells (CD45+).
