# Supplementary material for: TIGIT expression in renal cell carcinoma infiltrating T cells is variable and inversely correlated with PD-1 and LAG3
Source: Cancer Immunol Immunother. 2024 Aug 6;73(10):192. doi: 10.1007/s00262-024-03773-8 (PMC11303630; doi:10.1007/s00262-024-03773-8)
Supplement: Supplementary file 2 — Supplementary file2 (DOCX 97 KB) [file 262_2024_3773_MOESM2_ESM.docx]

**Supplementary Table**

| **Table 1:**  **Cox Proportional Hazards Model** |
| --- |
| 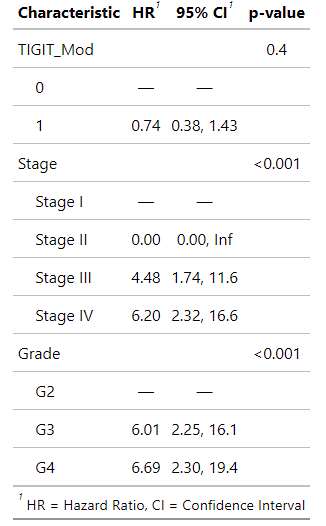 |
| **Table 1:** Cox proportional hazards model analyzing multivariable effects on 5-year survival from TCGA PanCancer Atlas ccRCC transcriptomic data. Variables include *TIGIT* expression (TIGIT_Mod) based on Z-score (low=0 and high=1), tumor stage and grade. Grade 1 was excluded from analysis due to low sample size and subsequent overfitting of the regression model. |
